# Multi-omics reveals the protective mechanisms of *Gastrodia elata* ethyl acetate extract in vascular dementia

**DOI:** 10.3389/fphar.2026.1630783

**Published:** 2026-01-23

**Authors:** Jie Tao, Tian Xiao, Zhuo Zhang, Jianghao Cheng, Jiaoyang Tan, Zhourong Zhao, Xiaohua Duan

**Affiliations:** Yunnan Key Laboratory of Dai and Yi Medicines, Yunnan University of Chinese Medicine, Kunming, China

**Keywords:** ethyl acetate extract, *Gastrodia elata*, inflammation, multi-omics research, vascular dementia

## Abstract

**Objective:**

This study aimed to investigate the ameliorative effect of the ethyl acetate extract of *Gastrodia elata* (EEGE) on vascular dementia (VD) and its underlying mechanisms.

**Methods:**

A VD rat model was established using the two-vessel occlusion method, while an *in vitro* cerebral ischemia injury model was constructed by subjecting HT22 cells to oxygen-glucose deprivation. The mechanisms were systematically explored through behavioral tests, ELISA, integrated network analysis, and combined metabolomic and transcriptomic techniques. Key targets were further validated by Western blot.

**Results:**

EEGE significantly improved cognitive function in VD rats. Integrated multi-omics and network analysis predicted that its effects involved two key targets, TNF and IGF1, and identified Parishin A and p-hydroxybenzaldehyde as prioritized drug metabolites for assessment. Subsequent experiments confirmed that EEGE effectively downregulated serum levels of IL-6, TNF-α, and IL-1β by modulating the IGF1-TREM2 signaling axis and the AMPK-SIRT1-FoxO1-NF-κB pathway.

**Conclusion:**

The improvement of cognitive dysfunction in vascular dementia by EEGE is closely associated with its regulation of the IGF1-TREM2 axis and the AMPK-SIRT1-FoxO1-NF-κB pathway, thereby mitigating neuroinflammation. This study provides experimental evidence and a potential mechanistic basis for further exploration of EEGE in VD intervention.

## Graphical Abstract

**Figure:**
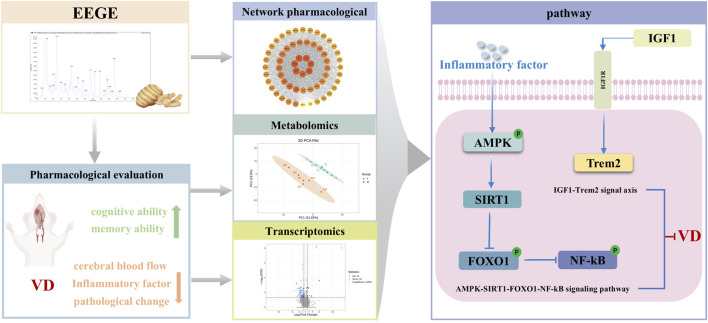

## 1 Introduction

Vascular dementia (VD), characterized as a syndrome of central nervous system impairment, is induced by cerebrovascular diseases that result in damage to brain cells, leading to a range of clinical manifestations, including cognitive impairment and memory loss (Pathan et al., 2024). It should be noted that most dementia patients have cerebrovascular diseases. VD accounts for about 15% of all dementia cases and is the second most common type of dementia (Wolters and Ikram, 2019). Vascular aging and the physiological decline of cognitive function caused by aging increase the risk of VD. Against the background of the increasing aging of the population, the incidence of VD in people over 60 years old in China has risen to 2.44%, and the number of patients is expected to quadruple by 2050 (Khan et al., 2020). Cognitive impairment associated with VD is relatively mild in the early stage and often difficult to detect. Usually within 6 months, patients will have cognitive dysfunction that gradually worsens and becomes increasingly difficult to reverse (Deardorff and Grossberg, 2019). As the disease progresses, the symptoms of late dementia are more obvious, which may be accompanied by changes in mood and personality (Morgan and Mc Auley, 2024). These changes will seriously affect the life, health and quality of life of patients, and at the same time put a huge burden on families and society.

At present, the main treatment methods used for VD are drug intervention, Including the use of cholinesterase inhibitors, such as donepezil and galantamine, as well as non-cholinergic medications, including memantine, nimodipine, and edaravone (Battle et al., 2021; Dang et al., 2024). These aforementioned therapeutic drugs are capable of rapidly improving the cerebral ischemia-hypoxia condition and modulating cerebral metabolic functions. However, they are associated with significant side effects, including nausea, vomiting, anorexia, diarrhea, and liver-kidney damage during their clinical application, and their long-term comprehensive pharmacological effects has been found to be unsatisfactory (Yang et al., 2024). Thus, there is a need to explore and develop safer and more effective drugs for intervention in VD.

Traditional Chinese medicine, known for its multi-component and multi-target nature, is recognized for its unique and significant advantages in the prevention and treatment of VD, offering substantial practical value and developmental potential (Bai and Zhang, 2021). *Gastrodia elata* (GE), a dried rhizome belonging to the orchid family, is widely used in treating cardiovascular and cerebrovascular diseases, as well as neuroprotection (Sun X. et al., 2023; Wu et al., 2023). Clinical evidence indicates that GE can markedly improve symptoms of senile dementia attributed to insufficient cerebral blood supply (Seok et al., 2019; Li et al., 2020; Wang T. et al., 2021; Luo et al., 2022; Huang et al., 2024). GE extract is known to confer anti-inflammatory, memory-enhancing, and nerve-protective capabilities (Lin et al., 2021; Yang and Tsai, 2022; Gong et al., 2024). Contemporary research points to a significant neuroprotective role of GE in ischemic brain injury. Its mechanism is mainly related to improving energy metabolism and inhibiting neuronal apoptosis (Zhang et al., 2024). The ethyl acetate extract of *G. elata* (EEGE) is regarded as a potential active component that plays an important role in ischemic brain injury. Existing studies have found that EEGE can effectively improve cerebral ischemic damage, thus playing a beneficial role in nerve protection. This effect is achieved through mechanisms such as vascular smooth muscle dilation and blood-brain barrier protection. It is known that EEGE can also protect neurons through antioxidant damage (Wang et al., 2024). Despite the established role of EEGE in preventing and treating ischemic cerebrovascular disease, its mechanism in the prevention and treatment of VD is not fully clear and needs further in-depth research and comprehensive clarification.

As an innovative research method, the integration of metabolomics and transcriptomics has been widely used in drug research to clarify the complex mechanism of drugs on organisms (Wu et al., 2019; Tian et al., 2024). This strategy involves comparing changes in endogenous metabolite levels between groups, so as to systematically evaluate and analyze the therapeutic effect of drugs and their mechanisms of action. Metabolic changes often cause corresponding intracellular gene expression adjustment, and changes in gene expression can reflect the physiological state of the organism (Chen et al., 2024). Therefore, the combined analysis of metabolomics and transcriptomics can comprehensively and systematically explore the mechanism of drug intervention in diseases, focusing on two key aspects: metabolites and gene expression. This two-pronged approach provides solid technical support and theoretical framework for modern traditional Chinese medicine research.

This study will use multi-group integration technology to jointly analyze the improvement mechanism of EEGE for VD. The study will replicate a rat model of two-vessel obstruction (2-VO) *in vivo*, and establish an HT22 cell damage model induced by hypoxic-glucose deprivation (OGD) *in vitro* to systematically assess the preventive and therapeutic effects of EEGE and its potentially active metabolites on VD. This study seeks to provide a theoretical basis for botanical drug applying GE to the treatment of VD, and is expected to provide new strategies and insights for its clinical management.

## 2 Materials and methods

### 2.1 Experimental animals and cell lines

The animals used in this experiment were obtained from Beijing SPF Technology Co., Ltd. (license number: SCXK (Jing) 2024-0001). The 60 male Sprague-Dawley rats required for this study were housed in a climate-controlled conditions are maintained at suitable parameters. This experiment was approved by the Animal Ethics Committee of Yunnan University of Chinese Medicine (Approval number: R-062019039). All experimental procedures were conducted in strict adherence to the policies of the National Institutes of Health.

Mouse hippocampal neuronal cells (HT22) were acquired from Shanghai Meiyan Biotechnology Co., Ltd. (CC-Y2137).

### 2.2 Preparation of experimental drugs

GE was purchased from Yunnan Huide Pharmaceutical Co., Ltd. and authenticated as genuine by Associate Professor Yin Zili from Yunnan University of Traditional Chinese Medicine. GE was weighed, crushed, and then refluxed three times with an appropriate amount of ethanol for 2 h each time. The three extracts were combined, heated to concentration, dissolved in deionized water, transferred to a reaction kettle, and extracted with ethyl acetate. After vacuum-drying, the EEGE was obtained.

### 2.3 Metabolite analysis of EEGE and network analysis

#### 2.3.1 Metabolite analysis of EEGE

Remove the specimen from the −80 °C ultra-low temperature freezer to thaw. Vortex for 1 min, proportionally add 70% methanolic water internal standard extraction solution pre-chilled at −20 °C (600 μL solution per 50 mg sample) and vortex for 15 min. Centrifuge (12,000 r/min, 4 °C) for 3 min, take the clear supernatant and filter it by a porous membrane (0.22 μm), then save it in the injection bottle for LC-MS/MS detection. The analysis detection was performed using an Ultra - Performance Liquid Chromatography - Electrospray Ionization Tandem Mass Spectrometry (UPLC - ESI - MS/MS) system, consisting of an ExionLC™ AD ultra - performance liquid chromatograph (SCIEX) and a triple quadrupole - linear ion trap hybrid mass spectrometer (SCIEX QTRAP® system). Liquid chromatographic separation was achieved on an Agilent SB - C18 reversed - phase column (1.8 µm, 2.1 × 100 mm) maintained at 40 °C in a column heater. The mobile phase comprised 0.1% formic acid in water (Phase A) and 0.1% formic acid in acetonitrile (Phase B), with the following gradient elution program: Phase B was linearly increased from 5% to 95% over 0–9 min, held at 95% for 1 min, then reduced back to 5% Phase B (95% Phase A) over 2 min, followed by a 2.9 min equilibration period. The flow rate was constant at 0.35 mL/min, and the injection aliquot was 2 µL.

The instrument operated in positive/negative ion switching mode, and its key operational conditions were as follows: ionization source temperature of 550 °C; curtain gas at 25 psi, nebulizer gas at 50 psi, and auxiliary gas at 60 psi. In multiple reaction monitoring mode, nitrogen was used as the collision gas (medium flow rate) for dynamic monitoring of characteristic ion pairs of metabolites during different elution intervals. The declustering potential and collision energy for each pair of ions were optimized and determined experimentally.

#### 2.3.2 Network analysis

Based on EEGE metabolite analysis, all detected substances were separately screened for active substances using three databases: TCMSP, SwissDrugDesign, and SymMap. In the TCMSP database, the screening criteria were set as oral bioavailability (OB) > 30 and drug-likeness (DL) > 0.18. In the SwissDrugDesign database, SwissADME (29 December 2025) was used for substance screening, with the requirement of high gastrointestinal absorption and at least two of the parameters of Lipinski, Ghose, Veber, Egan, and Muegge reaching “YES”. Then, the remaining metabolites were further screened using the Lipinski’s rule of five and the pan-assay interference compound (PAINS) filters from the PubChem database to exclude PAINS. Finally, the potentially active metabolites that satisfied the criteria were imported into the SwissTargetPrediction and SymMap databases for target prediction. Targets with a possibility > 0 were selected and after removing duplicate values, the remaining were the targets corresponding to potentially active metabolites in EEGE. Disease-related targets were selected using the DisGENET, TTD, and OMIM databases, and targets were retained after removing duplicate values. The intersection of disease-related targets and drug potentially active metabolite targets was taken and imported into the Cytoscape software. The main core targets were selected according to degree value. All results derived from this network analysis are based on predictive information from public databases and carry inherent risks of false positives and false negatives. These findings should be viewed as a starting point for generating experimental hypotheses rather than definitive pharmacological conclusions.

According to the screened core targets, molecular docking can be used for further assessment. Firstly, the 3D structure of metabolites was obtained from PubMed database, and the 3D structure of core protein was obtained from PDB database. The structure of protein receptor was pretreated by Py MOL software to remove water molecules and irrelevant original ligands. Finally, the CB-DOCK2 website is used to predict the binding sites of key active metabolites and targets and complete molecular docking, and Py MOL is used to visualize the docking results.

#### 2.3.3 Qualitative analysis of potentially active metabolites in EEGE

Combined with previous research, p-hydroxybenzaldehyde (AB2539), Parishin A (AB0475, Chengdu Alpha Biotechnology Co., Ltd., Chengdu, China) was selected for qualitative analysis. After precisely weighing 100 mg of the test sample of EEGE in a 5 mL volumetric flask, 80% methanol (C0690110225, Nanjing Chemical Reagent Co., Ltd., Nanjing, China) was added to the volume marked, and the solution was ultrasonicated for 30 min. After complete dissolution, the concentration of EEGE of the solvent was 20 mg/mL. Then, after precisely weighing 50 mg of the reference substance, place it in a 5 mL volumetric flask and add 80% methanol to the marked volume. After complete dissolution, the concentration of the reference substance was 1 mg/mL, which was then diluted with 80% methanol. Finally, 1 mL of both the reference substance and the test sample were added to a syringe, filtered through a 0.45 μm microporous membrane, and measured according to the chromatographic conditions (Agilent 1260 Infinity III, Agilent Technologies, Inc., CA, United States).

The chromatographic column model used was Agilent 5 TC-C18 (2) 250 × 4.6 mm (588925-902, Agilent Technologies, Inc., CA, United States). Acetonitrile (34851, Merck KGaA, Darmstadt, Germany) and water were used as mobile phase. The following methods were strictly followed in the separation process: from 0 to 25 min, acetonitrile (A) varied from 13% to 87%, and water (B) varied from 87% to 42%. The flow rate was adjusted to 1 mL/min, the analytical column temperature was controlled at 25 °C, the detector wavelength was selected as 270 nm, and a sample injection volume of 10 µL was employed.

### 2.4 Pharmacological effects evaluation of EEGE on 2-VO rats

#### 2.4.1 Animal grouping and administration

Following a 7-day adaptation phase, rats were randomly divided into the sham, model, memantine (0.9 mg/kg), high-dose EEGE (102 mg/kg), and low-dose EEGE (34 mg/kg) groups. Both the model and the sham groups received the same amount of normal saline. The memantine and the drug treatment groups received continuous intragastric administration of the drug or memantine after surgery, for a duration of 28 days.

#### 2.4.2 Animal model replication and evaluation

The sham group did not receive treatment, whereas the model group and the drug-administered groups replicated the VD rat model. An approximately 1.5-cm long longitudinal incision was made along the midline of the neck. The common carotid artery was located and the vagus nerve was dissected. The left and right common carotid arteries were surgically tied with sutures. First, the end farthest from the heart was ligated, followed by the end closest to the heart. The skin was then sutured.

The Zea Longa 5-level scoring method was used to evaluate the neurobehavioral scores of the modeled rats. The specific scoring criteria were as follows: Rats with 0 points showed no symptoms of neurological deficit; those with one point could not fully extend the right or left forelimb; those with two points circle to the right or left; those with three points tilt to the right or left; and those with four points lose the ability to walk. Rats with scores of 4 or 0 were excluded, and the remaining rats that met the criteria were used for subsequent experiments.

The cerebral blood flow of the rats that met the model criteria was measured. Rats were anesthetized by inhalation of 2% isoflurane (Veterinary Drug Approval Number of the State-Veterinary Drug No. 153717015, Qingdao Orbiepharm Co., Ltd., Qingdao, China). The rat’s head was fixed, and the hair on the top of the head near the front of the ear was completely removed. The area was disinfected with iodine tincture, and the skin on the top of the head was cut open about 1.5–2.0 cm with ophthalmic scissors. Taking the bregma and lambda points of the skull as the length, and a width of about 0.5 cm on both sides of the line connecting the two points as the observation range, with the optimal observation thickness being when blood vessels could be seen through the skull with the naked eye. A cotton ball dipped in 0.9% sham saline was gently applied to the exposed skull to mark the detection position. The rat was placed under a laser Doppler flowmeter, and imaging of cerebral blood flow changes was recorded. One area of the same size was selected from each of the left and right brains, and the average value of the two areas was calculated as the final cerebral blood flow change value.

#### 2.4.3 Morris water maze experiment

Prior to the experiment, the water temperature needs to be heated to 25 °C. An escape platform is placed in the third quadrant, and black pigment is added to the water to eliminate visual interference. The water maze experiment consists of a place navigation trial and a spatial probe trial. A 4-day place navigation training is conducted, with each training session set to 90 s. If a rat locates the platform within 90 s, the actual time taken is recorded as its escape latency. If a rat fails to find the platform within 90 s, the escape latency is recorded as 90 s, and the rat is then guided to stay on the platform for 15 s of learning. On the fifth day, the escape platform is removed, the trial time is extended to 120 s, and the number of times the rat crosses the former platform location is recorded to assess its memory retention.

#### 2.4.4 Hematoxylin-Eosin (HE) staining

For HE staining, the intact brain tissue was extracted from the rats and fixed in a 10% formaldehyde solution. Subsequently, the tissue was dehydrated through an alcohol gradient, cleared with xylene, and embedded. It was then sectioned into thin slices of 3 µm. The sections were stained with hematoxylin staining solution for 15 min. After washing, differentiation, and further staining, the slices were observed under an optical microscope, photographed, and analyzed.

### 2.5 Integrated network analysis of transcriptomics and metabolomics

#### 2.5.1 Metabolomics

Approximately 100 mg of brain tissue specimens was weighed from each group and triturated at a low temperature (4 °C) to prepare homogenates. The samples underwent a brief centrifugation (3,000 × g, 30 s). Following this, 400 μL of an ice-cold 70% methanol-in-water was added. After vortex oscillation (1,500 rpm, 5 min), samples were incubated in ice-bath standing for 15 min, followed by centrifugation at 12,000 × g for 10 min. We then transferred 300 μL of the clarified supernatant into an LC-MS vial for testing. We prepared quality control samples in parallel by pooling the extraction solutions from all experimental groups to assess the method’s repeatability. A hierarchical analysis approach is adopted to integrate multivariate statistical analyses and metabolic pathway enrichment analysis to systematically dissect the regulatory networks of potential biomarkers. Screening of differential metabolites was performed using VIP > 1 and P < 0.05.

Dual-mode detection was utilized for the electrospray ionization source, with its ionization chamber temperature held constant at 500 °C. The parameters for the ion transmission system were set as follows: spray voltage of 5,000 ± 500 V, nebulizing GSI of 55 psi and GSII pressure of 60 psi. The collision-induced dissociation parameters were configured in high-intensity mode.

A Waters ACQUITY UPLC HSS T3 C18 column was employed. We used a mobile phase of (A) 0.1% formic acid in water and (B) acetonitrile, with gradient elution according to the following program: the proportion of Phase B was linearly increased from 5% to 90% within 0–11.0 min, held at 90% until 12.0 min, then restored to 95% Phase A, followed by a 2.0-min equilibration period to complete method reset. Adjust the parameters to column temperature 40 °C, flow rate 1 mL/min and sample volume 2 μL.

#### 2.5.2 Transcriptomics

A 100 mg tissue sample from each group of rates was placed on dry ice. After grinding and centrifugation, 1 mL of Trizol was added. After the samples were centrifuged (12,000 rpm, 10 min, 4 °C), we collected 400 μL of the clarified supernatant into a new EP tube. Add isopropanol to the supernatant at a ratio of 1:1 (v/v), mix well by shaking for 15 s. Centrifuge at 12,000 rpm for 10 min at 4 °C. After discarding the supernatant, the precipitate was washed twice with 75% ethanol. The sample was then subjected to centrifugation (12,000 rpm, 1 min, 4 °C) to ensure complete removal of the supernatant. Finally, 50 μL of RNase-free water was added to dissolve the pellet.

After extracting suitable RNA, mRNA was extracted from total RNA, and was used as a template to reverse-transcribe and synthesize single-strand cDNA. Next, cDNA fragmentation, end-repair, and adapters were added to construct a cDNA library suitable for sequencing. The constructed library was placed in a sequencer for sequencing.

#### 2.5.3 Integrated network pharmacological analysis

The MetaboAnalyst 5.0 tool was used to analyze the differentially enriched metabolites and key gene pathways to evaluate the potential mechanism of EEGE in VD.

#### 2.5.4 Biochemical index detection

Remove the pre-inactivated serum samples from the −80 °C freezer and thaw them to a liquid state at room temperature. In strict compliance with the ELISA instruction manual, the concentrations of IL-6 (RA20607, Bioswamp Co., Ltd., Wuhan, China), TNF-α (RA20035, Bioswamp Co., Ltd., Wuhan, China), and IL-1β (RA20020, Bioswamp Co., Ltd., Wuhan, China) were detected. In the microplate reader, the zero point was set to zero with the blank control well at 450 nm and then the OD value was measured. A standard curve was created based on the convergence and OD values of the standard products. We calculated the concentrations by inputting the measured values into the standard curve equation.

#### 2.5.5 Western blot (WB) detection

The brain tissues of each group were thawed and RIPA lysis buffer (KGP702-100, Jiangsu KeyGEN BioTECH Corp., Ltd. Jiangsu, China) containing PMSF (97064-898, Amresco, VWR International, OH, United States) was added. A 10% separating gel and a 5% stacking gel, appropriate for the protein molecular weight range, were prepared for SDS-PAGE electrophoresis. An appropriate amount of pre-cooled 1× electrophoresis buffer was added to adjust the sample loading amount to 60 μg. After adjusting the voltage, the electrophoresis was ended when the target band reached an appropriate position. After completing the constant-voltage membrane transfer, the membrane was incubated in Ponceau S staining solution and washed twice with TBST (G0001, Wuhan Servicebio Biotechnology Co., Ltd., WuHan, China). Transfer the membrane to a Petri dish containing blocking solution, and shake it on a shaker maintained at RT for a 1-h period. Wash the membrane and perform blocking. Add the primary antibody diluted with TBST, and the dilution ratios are shown in Table 1. Incubate overnight at 4 °C. Then wash and re-block the membrane. Add the diluted secondary antibody, with the dilution ratios shown in Table 1. After room temperature for 1 h, wash the membrane. Finally, add equal volumes of luminescent rea-gent A and reagent B, and detect using the Tanon 5200 chemiluminescence imaging workstation. Analyze the optical density values of the protein expression levels using Image Pro Plus 6.0.

**TABLE 1:** Antibody information.

Antibodies	Dilution (application)	Catalog number	Company name
IGF-1	1:500	DF6096	Affinity Biosciences Pty Ltd., Melbourne, Australia
Trem2	1:1,000	PA5-87933	Thermo Fisher Scientific Inc., MA, United States
FoxO1	1:1,000	14952	Cell Signaling Technology, MA, United States
Phospho-FoxO1 ^(Ser256)^	1:1,000	9461	
NF-kB p65	1:1,000	8242	
Phospho-NF-κB p65 ^(Ser536)^	1:1,000	3033	
Phospho-AMPKα ^(Thr172)^	1:1,000	2535	
AMPK	1:1,000	2532	
SIRT1	1:1,000	8469	
β-actin	1:1,000	ab8227	Abcam plc, Cambridge, United Kingdom
Goat Anti-GEbbit IgG H&L (HRP)	1:10,000	ab6721	
GEbbit anti-mouse IgG H&L (HRP)	1:10,000	ab6728	

Antibodies	Dilution (application)	Catalog number	Company name
IGF-1	1:500	28539-1-AP	Proteintech, Wuhan, China
Trem2	1:1,000	PA5-87933	Thermo Fisher Scientific Inc., MA, United States
FoxO1	1:1,000	14952	Cell Signaling Technology, MA, United States
Phospho-FoxO1 ^(Ser256)^	1:1,000	9461	
NF-kB p65	1:1,000	10745-1-AP	Proteintech, Wuhan, China
Phospho-NF-κB p65 ^(Ser536)^	1:1,000	80379-1-AP	
Phospho-AMPKα ^(Thr172)^	1:1,000	2535	Cell Signaling Technology, MA, United States
AMPK	1:1,000	2532	
SIRT1	1:1,000	8469	
β-actin	1:1,000	7967	
Goat Anti-GEbbit IgG H&L (HRP)	1:10,000	ab6721	Abcam plc, Cambridge, United Kingdom
GEbbit anti-mouse IgG H&L (HRP)	1:10,000	ab6728	

### 2.6 Experimental assessment *in vitro*

#### 2.6.1 Cell grouping, drug administration, and establishment of the OGD model

HT22 cells were divided into the control group, OGD group, and drug-administered group. Cells in the control group were not treated. For the OGD group, an *in vitro* OGD model was established by culturing the cells in a sugar-free DMEM medium (MA0212, Dalian Meilun Biotechnology Co., Ltd., Dalian, China) containing 6 mmol/L Na_2_S_2_O_4_ (S433998, Shanghai Aladdin Biochemical Technology Co., Ltd., Shanghai, China) for 2 h. The corresponding concentration of the drug was added 24 h before OGD, which continued until the end of OGD.

#### 2.6.2 MTT cell viability assay

To determine the effective concentration range of the drug, different concentrations of p-hydroxybenzaldehyde (5, 10, 20, 40, 80 μg/mL),Parishin A (20, 40, 80, 160, 320 μg/mL) and EEGE (10, 20, 40, 60, 180 μg/mL) were co-incubated with OGD-treated HT22 cells for 24 h. Preparation of the drug stock solution (10 mg/mL) was as follows: First, a quantity of 100 mg EEGE was weighed and dissolved in 0.1 mL of DMSO. This was followed by dilution with 9.9 mL of PBS and filtration through a 0.22 μm sterilizing membrane. The stock solution was further diluted with complete culture medium to the desired concentrations for treating the cells. To determine cell viability, the MTT assay was performed. After the treatment period, we added 20 μL of MTT solution (5 mg/mL) to each well and continued incubation in the dark for 4 h. Subsequently, we removed the supernatant from each well, added 150 μL of DMSO, and placed the plates on a shaker at 37 °C for 10 min to fully dissolve the formazan deposits. The absorbance was then read at 490 nm on a plate reader.

### 2.7 Western blot (WB) detection

Protein samples from the HT22 cell were lysed using RIPA lysis buffer supplemented with protease inhibitors and phosphatase inhibitors. After processing of the sample, the protein concentration was quantified using the BCA method. Equal amounts of protein samples were denatured with loading buffer, separated by SDS-polyacrylamide gel electrophoresis, and transferred to polyvinylidene fluoride membranes. The membranes after transfer were blocked with 5% skimmed milk. Transfer the membrane to a Petri dish containing blocking solution, and shake it on a shaker maintained at RT for a 1-h period. Wash the membrane and perform blocking. Add the primary antibody diluted with TBST, and the dilution ratios are shown in Table 2. Before incubating overnight at 4 °C. Subsequently, the HRP-labeled anti-immunoglobulin (Ig) G secondary antibody was incubated at room temperature for 1 h. After thorough washing, the immune reaction bands were displayed using enhanced chemiluminescence reagents and images were captured by a chemiluminescence imaging system. Finally, the gray values of the target bands were quantitatively analyzed using ImageJ software, and the expression of the target proteins were standardized with β-actin as the internal reference.

**TABLE 2:** Standard - compliant Metabolites.

Metabolites	Formula	CAS
Isonicotinic acid	C_6_H_5_NO_2_	55-22-1
Methyl L-pyroglutamate	C_6_H_9_NO_3_	4931-66-2
D-pantothenic acid	C_9_H_17_NO_5_	79-83-4
Confertifoline	C_15_H_22_O_2_	1811-23-0
Benzaldehyde	C_7_H_6_O	100-52-7
Dehydroabietic acid	C_20_H_28_O_2_	1740-19-8
3-Hydroxycinnamic acid	C_9_H_8_O_3_	14755-02-3
Butyl isobutyl phthalate	C_16_H_22_O_4_	17851-53-5
α-Hydroxycinnamic acid	C_9_H_8_O_3_	5801-57-0
Diisobutyl phthalate	C_16_H_22_O_4_	84-69-5
Senkyunolide B	C_12_H_12_O_3_	93236-67-0
5-Hydroxymethylfurfural	C_6_H_6_O_3_	67-47-0
Syringic acid	C_9_H_10_O_5_	530-57-4
2-Hydroxycinnamic acid	C_9_H_8_O_3_	583-17-5
Salicylic acid	C_7_H_6_O_3_	69-72-7
6-Hydroxynicotinic acid	C_6_H_5_NO_3_	5006-66-6
4-Nitrophenol	C_6_H_5_NO_3_	100-02-7
Choline	C_5_H_14_NO_+_	62-49-7
2-Decanol	C_10_H_22_O	1120-06-5
Histidinol	C_6_H_11_N_3_O	501-28-0
4-Hydroxybenzaldehyde	C_7_H_6_O_2_	123-08-0
4-Hydroxybenzoic acid	C_7_H_6_O_3_	99-96-7
2,4-Di-Tert-Butylphenol	C_14_H_22_O	96-76-4
Lumichrome	C_12_H_10_N_4_O_2_	1086-80-2
Vanillic acid	C_8_H_8_O_4_	121-34-6
2-Phenyloxirane	C_8_H_8_O	20780-54-5
Phthalic acid	C_8_H_6_O_4_	88-99-3
Phenyl acetate	C_8_H_8_O_2_	122-79-2
4-Hydroxyacetophenone	C_8_H_8_O_2_	99-93-4
2-Methoxy-4-ethenylphenol	C_9_H_10_O_2_	7786-61-0
Zarzissine	C_5_H_5_N_5_	160568-14-9
3-[(1-Carboxyvinyl)oxy]benzoic acid	C_10_H_8_O_5_	16929-37-6
Polygodial	C_15_H_22_O_2_	6754-20-7
2-(Formylamino)benzoic acid	C_8_H_7_NO_3_	3342-77-6
Parishin A	C_45_H_56_O_25_	62499-28-9
N-(acetyl)phenylalanine	C_11_H_13_NO_3_	2018-61-3

### 2.8 Data statistics

All data were analyzed using GraphPad Prism 9.4.1 software. Experimental results were expressed as the mean standard deviation (SD) of at least three independent experiments. The following methods were used to test statistical significance, or analysis of variance was performed when comparing more than two groups. A P-value<0.05 was considered to indicate a significant difference.

## 3 Results

### 3.1 Identification of metabolites in EEGE

**FIGURE 1:**
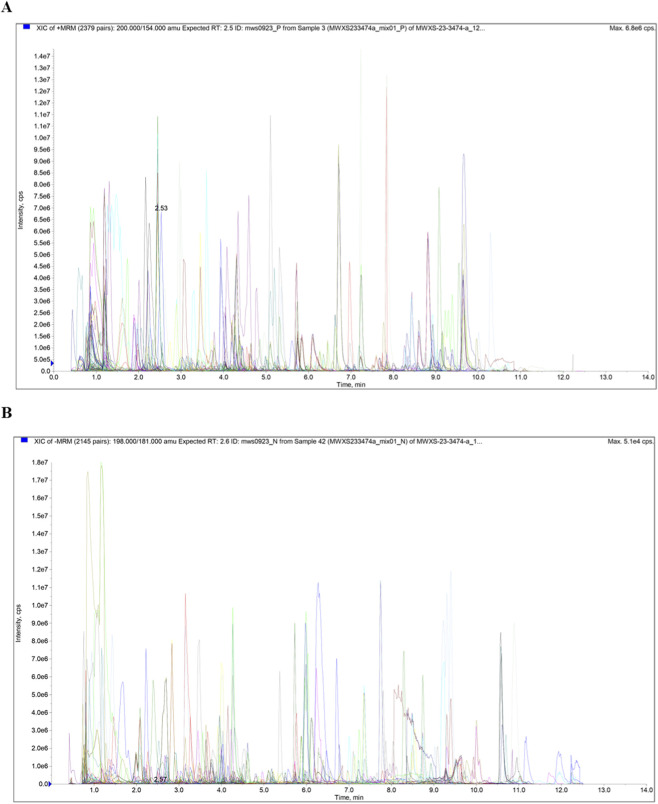
Extracted ion chromatogram (XIC) in multiple reaction monitoring (MRM) mode. **(A)** XIC of P. **(B)** XIC of P. Abbreviation: N, negative ion mode; P, positive ion mode.

A total of 987 metabolites were identified. These metabolites include 181 phenolic acid metabolites, 125 amino acids and their derivatives, 123 lipid metabolites, 100 alkaloid metabolites, 89 organic acid metabolites, 76 flavonoid metabolites, 44 nucleotide and their derivatives, 37 lignin and coumarin metabolites, 34 terpenoid metabolites, 19 quinone metabolites, 5 tannin metabolites, 3 steroid metabolites, and 151 other metabolites. The multi-peak graphs of the EEGE samples are shown in Figures 1A,B.

### 3.2 Analysis of network analysis results

**FIGURE 2:**
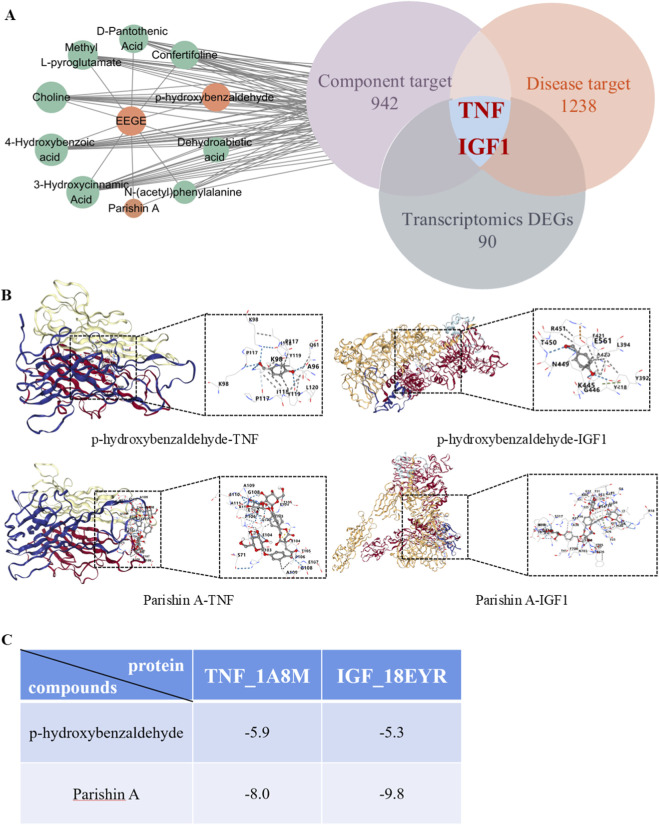
Network Analysis. **(A)** Top 10 metabolites and core target map of EEGE degree value. **(B)** Molecular docking diagram of metabolites and targets. **(C)** Molecular docking binding energy.

After screening, a total of 36 metabolites met the criteria (Table 2). Combining with the prediction results of Swiss target prediction, 942 metabolite targets were obtained, and 1,238 disease-related targets were retrieved through search. Taking the intersection of the differentially expressed genes in transcriptomics, two key targets TNF and IGF1 were obtained (Figure 2A). Using Parishin A and p-hydroxybenzaldehyde as ligands and TNF and IGF1 as receptors, the binding energy was calculated. Generally, it is considered that a binding energy less than −5.0 kcal/mol indicates a good binding effect between the metabolite and the target. The binding energy results showed that the metabolites and targets screened in this study all had good binding activity (Figures 2B,C).

### 3.3 Qualitative analysis of potentially active metabolites in EEGE

**FIGURE 3:**
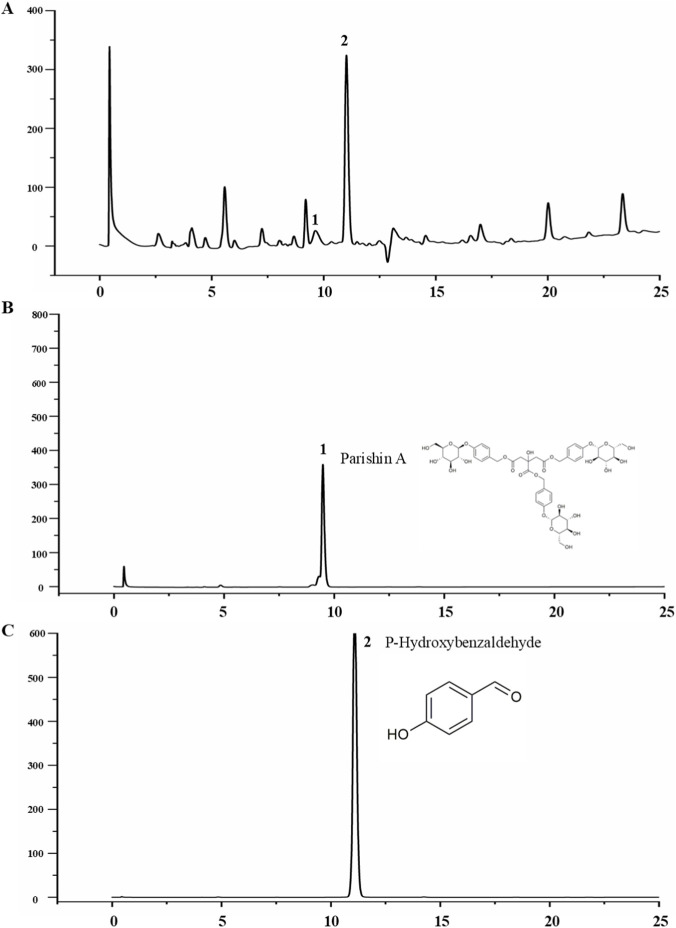
HPLC. **(A)** Test sample solution. **(B)** Reference substance solution, one parishin A. **(C)** Reference substance solution, two p-hydroxybenzaldehyde.

Based on network pharmacological predictions and literature reports, we focused on three potential active metabolites related to neuroprotective effects in EEGE: Parishin A and p-hydroxybenzaldehyde. The results showed that Parishin A and p-Hydroxybenzaldehyde exhibited peaks at 9.20 and 11.00 s respectively, while the corresponding peaks of the test samples appeared at 9.487 and 11.08 s. The retention time of the test sample is within ±0.5 min, indicating good specificity (Figures 3A–C).

### 3.4 Pharmacological effects evaluation of EEGE in VD rats

**FIGURE 4:**
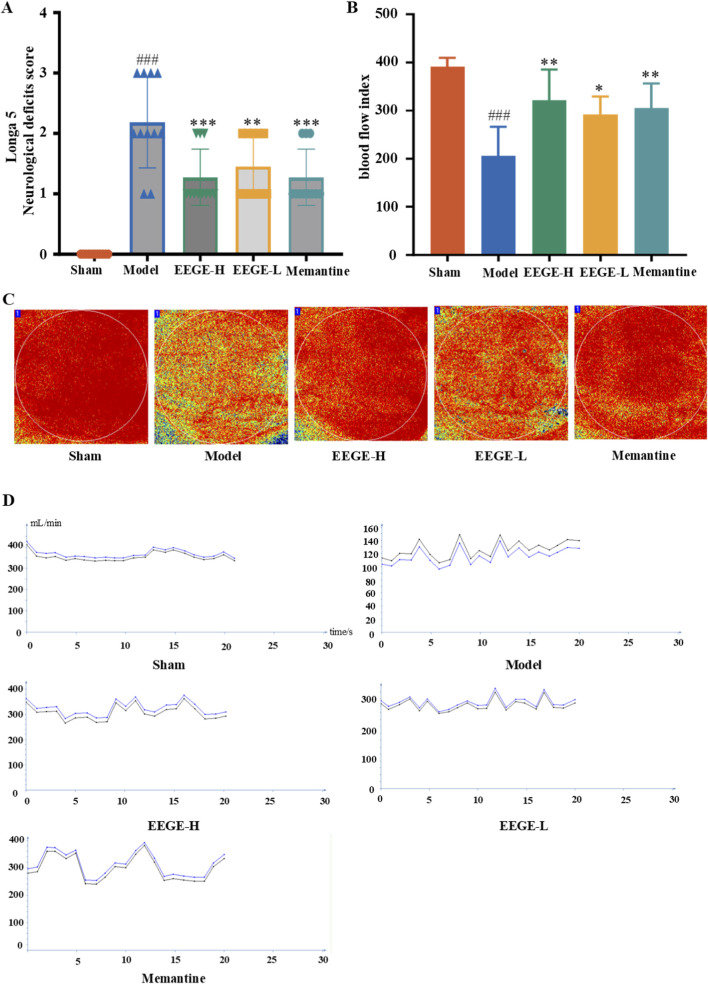
Evaluation of the VD model and the pharmacological effects of EEGE. **(A)** Scores of the Zea-Longa Neurological Behavioral Assessment Method (n = 8). **(B, C)** Changes in the mean cerebral blood flow of rats in each group (n = 6). **(D)** Alterations in cerebral blood flow over a 20s period for rats in each group (n = 6). vs. sham group, ^###^P < 0.001; vs. model group, *P < 0.05 and **P < 0.01.

After modeling, the neurological function scores of the administration groups showed a significant downward trend (P < 0.01, Figure 4A). The model group also showed a marked decrease in cerebral blood flow relative to the Sham group (P < 0.001). Together, the neurological scores confirmed the successful replication of the VD model. EEGE administration significantly increased cerebral blood flow in both dose groups compared to the model group (P < 0.01 and P < 0.05, Figures 4B–D). These results suggest that EEGE intervention has a certain positive effect on improving nerve damage and cerebral blood flow.

**FIGURE 5:**
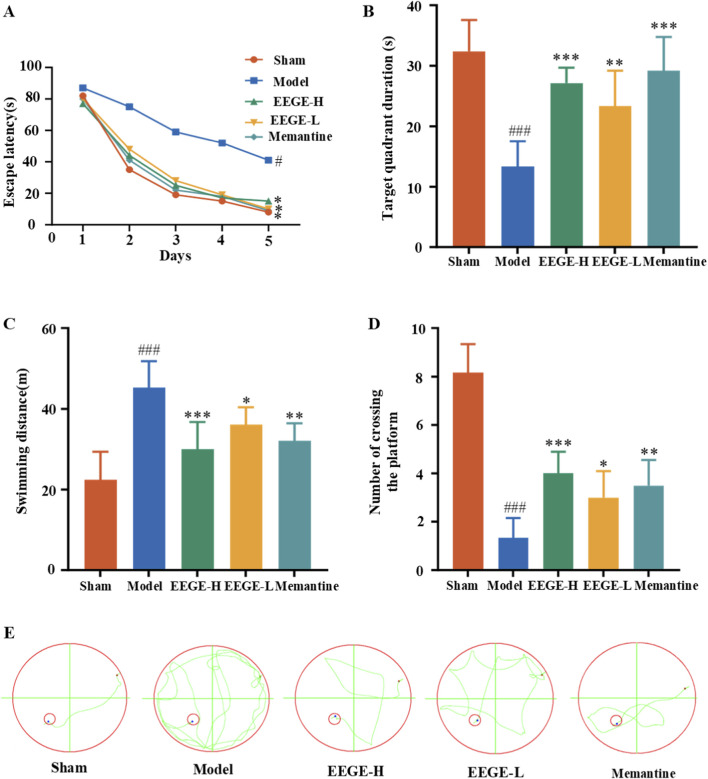
Evaluation of cognitive memory ability. **(A)** During the 5-day route planning exercise, the measurement departure delay was controlled within 90 s. **(B)** Percentage of residence time in the target quadrant within 120 s. **(C)** Swimming distance within 120 s. **(D)** Crossings across the target platform area. **(E)** Representative navigation traces from the spatial probe test. The starting position (red dot), end point (blue dot), and target platform (red circle) are indicated. Presented as mean ± SD (n = 6). vs. sham group, ^###^P < 0.001; vs. model group, *P < 0.05, **P < 0.01.

Morris water maze experiment showed that the escape latency of model rats was significantly longer than that of sham group. Indicating impaired learning and memory abilities in the model group rats. Compared with the model group, the drug administration group significantly shortened the escape latency of VD rats (Figure 5A). In the spatial exploration experiment on day 5, the residence time in the target quadrant and the number of crossings through the target quadrant of VD rats were significantly lower than those of the sham group (P < 0.001), while the swimming distance within 120 s was significantly higher than that of the sham group (P < 0.001). Compared with the model group, Significant reversals were achieved after drug intervention (P < 0.01, P < 0.05, Figures 5B–D). The swimming trajectories of rats in the sham group showed obvious regularity and target-orientation. The swimming trajectories of VD rats were rather chaotic and mostly random. Although the swimming trajectories of rats in the administration groups showed a certain degree of randomness, they generally showed a trend approaching that of the sham group (Figure 5E). This indicates that EEGE can alleviate the cognitive dysfunction of VD rats.

**FIGURE 6:**
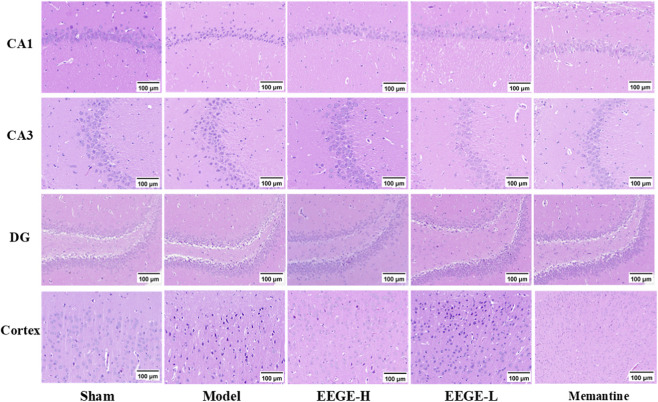
Changes in pathological staining of CA1, CA3, DG regions and cortex of VD rats treated with EEGE (n = 3).

The results of HE staining showed that in the brain sections of rats in the sham group, the CA1, CA3, DG regions, and the cerebral cortex were arranged neatly with complete structures and no obvious pathological changes. In the model group, due to cerebral ischemia, nerve cells were swollen, and the cell nuclei were pyknosed and showed vacuolar changes. The above-mentioned lesions in rats of the EEGE group were significantly improved (Figure 6).

### 3.5 Metabolomic analysis results of VD rats treated with EEGE

**FIGURE 7:**
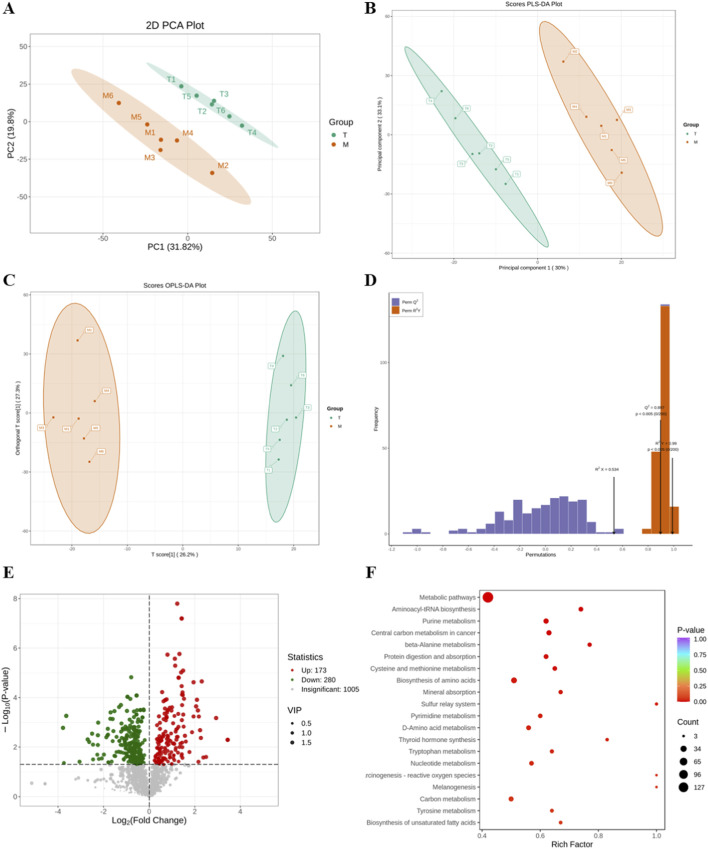
Metabolomics and transcriptomics analysis. **(A)** PCA score plot. **(B)** PLS-DA score plot. **(C)** OPLS-DA score plot. Each point represents an individual sample. Samples from the same group are displayed in the same color: orange dots represent the Model group, and green dots represent the Drug-administered group. **(D)** OPLS-DA assessment plot. The orange dots represent the R^2^Y of the permuted models, and the purple dots represent the Q^2^ of the permuted models. The black arrows indicate the values of R^2^X, R^2^Y, and Q^2^ of the original model. The original model was considered valid and excellent, as evidenced by a Q^2^ value of 0.897 (greater than the threshold of 0.5) and all P-values <0.05. **(E)** Volcano plot of differential metabolism. Each point represents a metabolite. Red points denote significantly upregulated metabolites, green points denote significantly downregulated metabolites, and gray points represent metabolites with no significant changes. **(F)** KEGG pathway enrichment analysis. The color of the points corresponds to −log_10_ (p-value), where a redder hue indicates a more significant enrichment. The size of the points represents the number of differential metabolites enriched in the pathway.

To investigate the changes of different metabolites between groups, the grouping was distinguished by the score plots of principal metabolite analysis (PCA) and partial least squares-discriminant analysis (PLS-DA). The PCA showed that the metabolic states of rats in the administration group and the VD model group were significantly separated, indicating a good grouping situation (Figure 7A). Orthogonal partial least squares-discriminant analysis (OPLS-DA), which combines PLS-DA, showed a clear distinction between groups. Additionally, OPLS-DA can assess the success of the model through the predictive parameters *R* ^2^ and Q^2^. The results showed that *R* ^2^ = 0.99 and Q^2^ = 0.897 (Figures 7B–D), and values close to one indicate an effective model. We identified 453 metabolites that were differentially expressed between the model group and the group treated with EEGE, and the volcano plot is shown in Figure 7E. Through analysis, 173 metabolites were upregulated and 280 metabolites were downregulated. These mainly included inosine, L-glutamyl-L-glutamine, γ-L-glutamyl-L-glutamine, α-D-ribose 5-phosphate, 7-hydroxy-6-methyl-8-ribosyl-dihydrotetrahydropteridine, arabinosylhypoxanthine, 2′-deoxyadenosine-5′-monophosphate, S-nitrosoglutathione, glutamyl-valine, ribose 1-phosphate, etc. To further clarify the potential mechanisms of the metabolites, the metabolites were imported into the KEGG database for pathway enrichment analysis. Finally, the top 20 pathways ranked by P-value were selected. The main metabolic pathways involved included metabolic pathways, carbon metabolism, etc. (Figure 7F). The metabolomic results indicated that some metabolites were re-versed after EEGE intervention.

### 3.6 Transcriptomic analysis results of VD rats treated with EEGE

**FIGURE 8:**
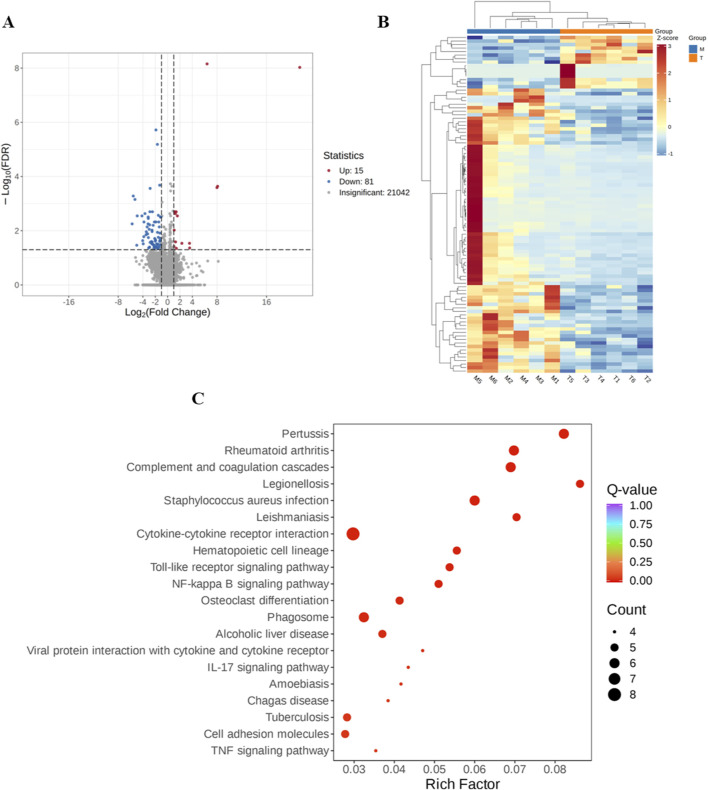
Analysis of transcriptome data. **(A)** Volcano plot highlighting gene expression changes. Significantly upregulated and downregulated genes are colored red and blue, respectively; genes without significant change are shown in gray. **(B)** Expression heatmap for the identified DEGs, with a red-blue color key for expression levels. **(C)** Results of KEGG pathway enrichment presented as a scatter plot. The dot size is proportional to the number of DEGs mapped to a pathway, and the red color intensity signifies the level of enrichment significance.

To clarify the neuroprotective action of EEGE, transcriptional profiling via RNA-seq was conducted to examine gene expression changes in the brains of VD rats administered with EEGE. A comparison between the model and treatment groups identified differentially expressed genes based on the criteria of |log_2_Fold Change| ≥ 1 and FDR < 0.05. In total, 96 genes showed significant expression changes, with 81 increased and 15 decreased, as presented in the volcano plot (Figure 8A). A heatmap demonstrates the expression dynamics of these genes, using a color scheme of red (elevated) and blue (reduced) (Figure 8B). To probe the potential functional mechanisms, enrichment analysis was carried out on the gene set, highlighting significant associations with pathways including Cytokine-cytokine receptor interaction, Phagosome, Neuroactive ligand-receptor interaction, Cell adhesion molecules, and the TNF signaling pathway (Figure 8C).

### 3.7 Integrated analysis of metabolomic and transcriptomic studies

**FIGURE 9:**
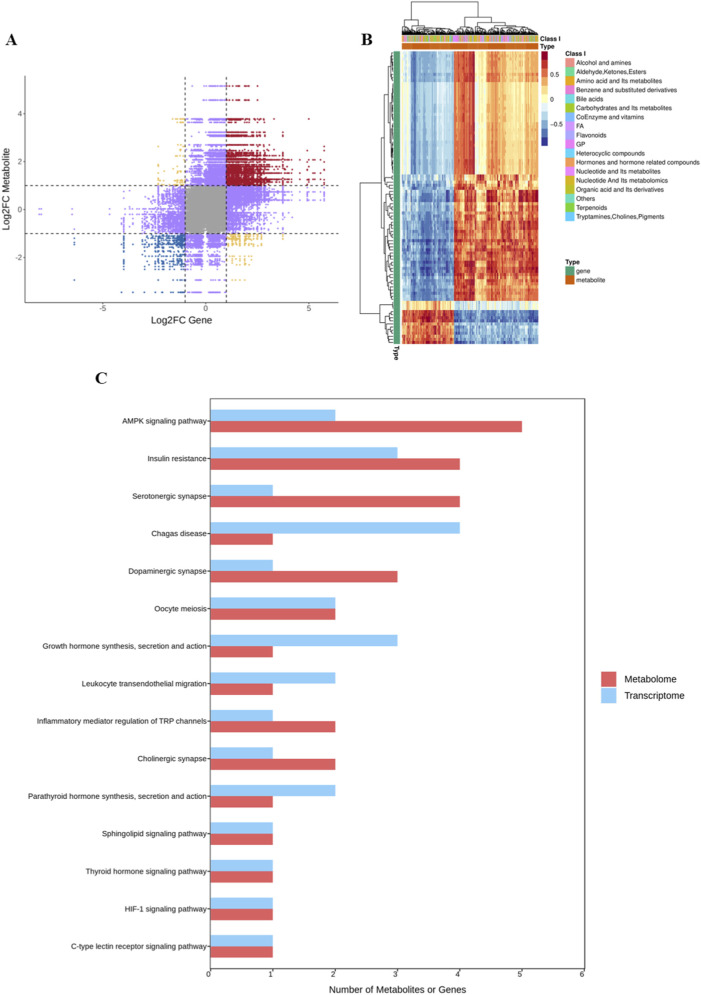
The integrated metabolomics and transcriptomics analysis. **(A)** A nine-quadrant plot depicting the correlation between gene and metabolite fold changes **(B)** A heatmap illustrating the correlation matrix between genes (rows) and metabolites (columns). The color scale represents correlation strength and direction (red: positive, blue: negative), while the top annotation bar colors distinguish the two experimental groups. **(C)** A horizontal bar plot displaying the number of omics features (metabolites in red, genes in green) enriched in significantly altered KEGG pathways.

Based on the differential metabolites and differential genes, the parts with a Pearson correlation coefficient absolute value greater than 0.8 and a p-value less than 0.05 were screened. The results of the nine-quadrant analysis based on the fold changes of gene and metabolite expression demonstrate that: the black dashed line in the figure divides the coordinate plane into nine regions. The central region (the fifth quadrant) indicates no significant differences in both; the third and seventh quadrants suggest a positive correlation between gene expression and metabolite levels; while the first and ninth quadrants show a negative correlation trend, implying a potential negative regulatory relationship (Figure 9A). The second, fourth, sixth, and eighth quadrants represent unchanged expression of metabolites and genes. A cluster heatmap was drawn according to the calculation results of differential genes and differential metabolites. Red indicates a positive correlation between genes and metabolites, and blue indicates a negative correlation (Figure 9B). Combining the results of transcriptomics and metabolomics, we found that the functional-related pathways of differential genes and differential metabolites mainly included mineral absorption, AMPK signaling pathway, alcoholism, insulin resistance, etc. (Figure 9C). Through the combined analysis, it was found that the expressions of IGF1 and FoxO in the AMPK signaling pathway were reversed.

### 3.8 Effects of EEGE on the contents of serum IL-6, TNF-α, and IL-1β in 2-VO rats

**FIGURE 10:**
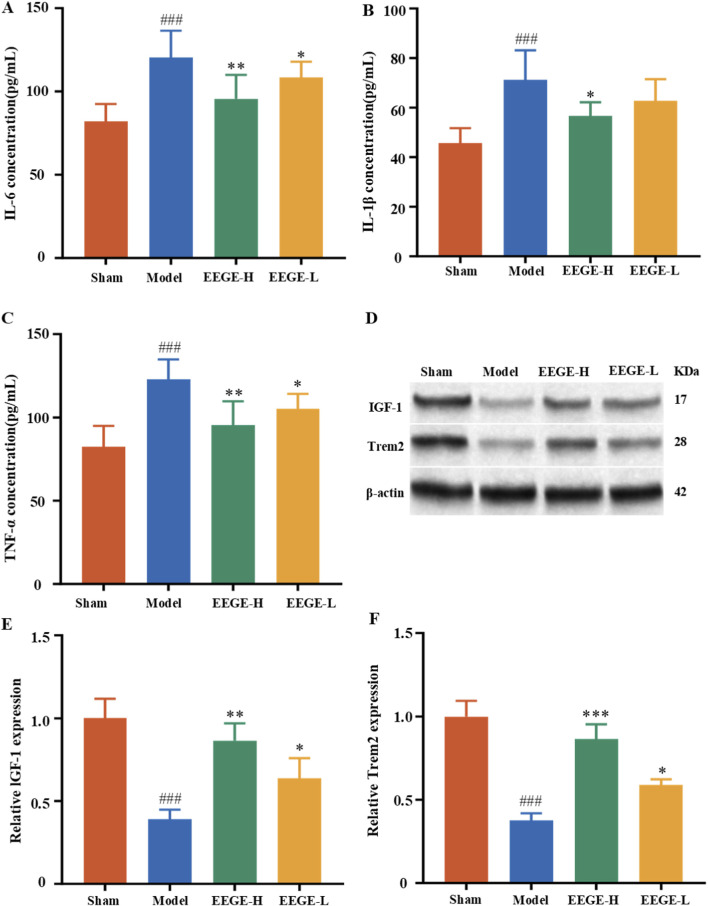
Assessment of systemic inflammation and the Trem2-IGF-1 signaling axis in the rat brain. The concentrations of **(A)** IL-6, **(B)** IL-1β, and **(C)** TNF-α in serum were measured (mean ± SD, n = 6). **(D)** Shows representative blot images. **(E, F)** Statistical results of IGF-1 and Trem2 expression levels presented as mean ± SD (n = 3, biological replicates). vs. sham group, ^###^P < 0.001; vs. model group, *P < 0.05, **P < 0.01.

To evaluate the mechanism of EGGE in regulating inflammation, the ELISA method was used to detect changes in the concentrations of pro-inflammatory cytokines IL-6, TNF-α, and IL-1β in the serum of rats in each group. Experimental data showed that compared with the sham group, the concentrations of IL-6, TNF-α, and IL-1β in the serum of rats in the VD model group were significantly increased (P < 0.001). Following EGGE intervention, the expression of inflammatory cytokines was significantly reversed (P < 0.001, P < 0.01, P < 0.05, Figures 10A–C).

### 3.9 Effects of EEGE on the IGF1-TREM2 and AMPK-SIRT1-FoxO1-NF-κB signaling pathways in 2-VO rats

We detected the expressions of key proteins in the IGF1-TREM2 signaling axis. The research results showed that, in comparison to the sham group, the protein levels of IGF-1 and Trem2 decreased (P < 0.01). In comparison to the model group, EEGE re-versed the expressions of IGF-1 and Trem2 (P < 0.01, P < 0.05, Figures 10D–F).

**FIGURE 11:**
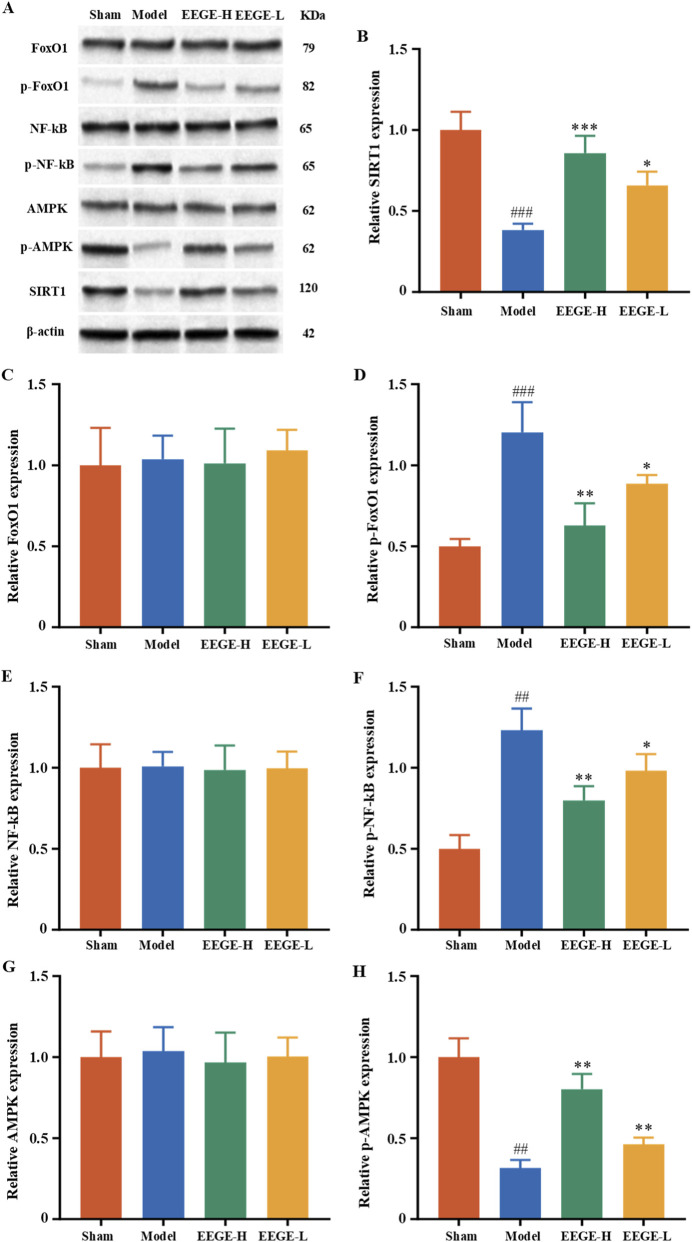
EEGE improves damage in VD rats via the AMPK/SIRT1/FoxO1/NF-κB pathway. The expression of p-FoxO1, FoxO1, p-NF-κB, NF-κB, p-AMPK, AMPK, and SIRT1 proteins in the hippocampal tissues of rats were detected by Western blotting. β-actin was used as the internal reference protein. **(A)** Protein band blot images of p-FoxO1, FoxO1, p-NF-κB, NF-κB, p-AMPK, AMPK, and SIRT1. **(B–H)** Statistical analysis of p-FoxO1, FoxO1, p-NF-κB, NF-κB, p-AMPK, AMPK, and SIRT1 proteins. There were no significant differences in non-phosphorylated proteins, and they were not statistically significant. For phosphorylated proteins. Presented as mean ± SD (n = 3, biological replicates). vs. sham group, ^##^P < 0.01; vs. model group, *P < 0.05, **P < 0.01.

The results of protein expressions in the AMPK-SIRT1-FoxO1-NF-κB signaling pathway showed that, in comparison to the sham group, the protein levels of p-FoxO1/FoxO1 and p-NF-κB/NF-κB increased, while the protein levels of p-AMPK/AMPK and SIRT1 decreased (P < 0.01). After administration, these protein levels were significantly restored (P < 0.01, P < 0.05, Figures 11A–H).

### 3.10 Effects of EEGE on the viability of HT22 cells

**FIGURE 12:**
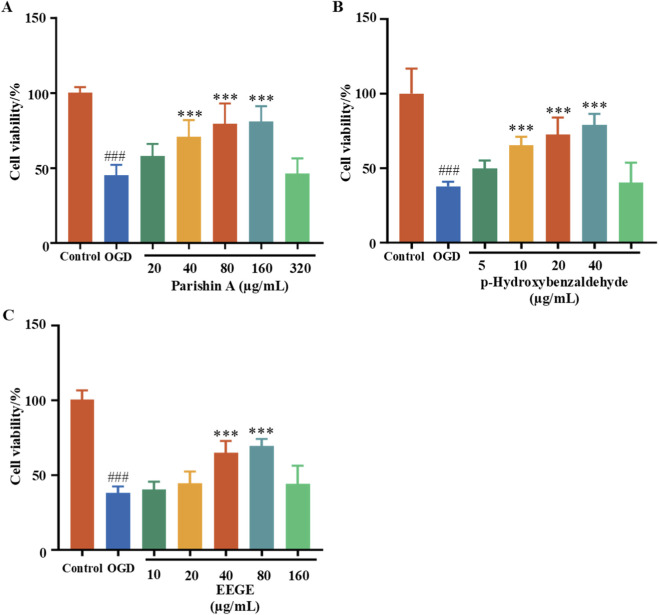
MTT assay for cell viability. Percentage of HT22 cell viability induced by OGD affected by **(A)** Parishin A, **(B)** p-hydroxybenzaldehyde and **(C)** EEGE. Presented as mean ± SD (n = 6). vs. sham group, ^###^P < 0.0 1; vs. model group, **P < 0.01, ***P < 0.001.

MTT assay results showed that the cell survival rate in the OGD group was less than 50% compared to the control group. In comparison to the OGD group, when the concentration of Parishin A reached 40, 80, and 160 μg/mL, the cell viability was approximately 61% (P < 0.001), 69% (P < 0.001), and 70% (P < 0.001), respectively; when the concentration of p-Hydroxybenzaldehyde reached 10, 20, and 40 μg/mL, the cell viability was roughly 65% (P < 0.001), 73% (P < 0.001), and 79% (P < 0.001), respectively; when the concentration of EEGE reached 40 and 80 μg/mL, the cell viability was approximately 65% (P < 0.001) and 70% (P < 0.001, Figures 12A–C). In conclusion, Parishin A, p-Hydroxybenzaldehyde, and EEGE can all significantly increase cell viability and exert protective effects on HT22 cells damaged by OGD. The drug concentration of EEGE that can significantly restore cell viability was selected for subsequent Western blot experiments.

### 3.11 Effects of EEGE on the IGF1-TREM2 and AMPK-SIRT1-FoxO1-NF-κB signaling pathways in HT22 cells

**FIGURE 13:**
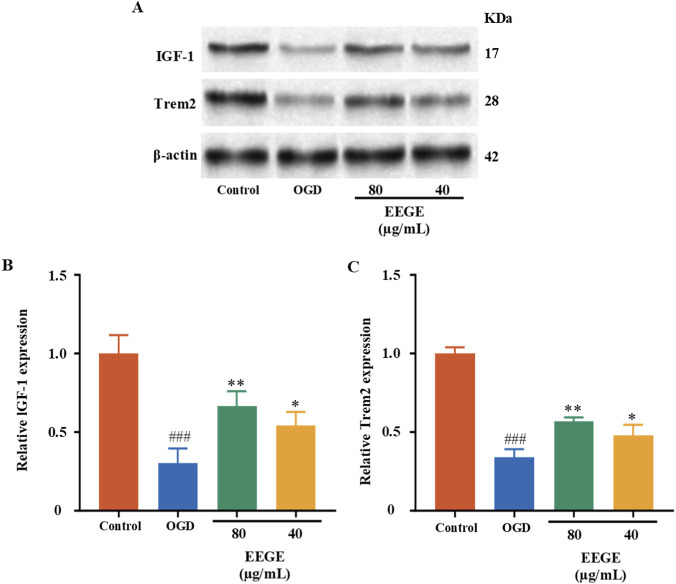
Western blot analysis of IGF-1 and Trem2 expression in HT22 cells (normalized to β-actin). **(A)** Representative blots. **(B, C)** Quantitative data for IGF-1 and Trem2, presented as mean ± SD (n = 3, biological replicates). vs. sham group, ^###^P < 0.001; vs. model group, *P < 0.05, **P < 0.01.

**FIGURE 14:**
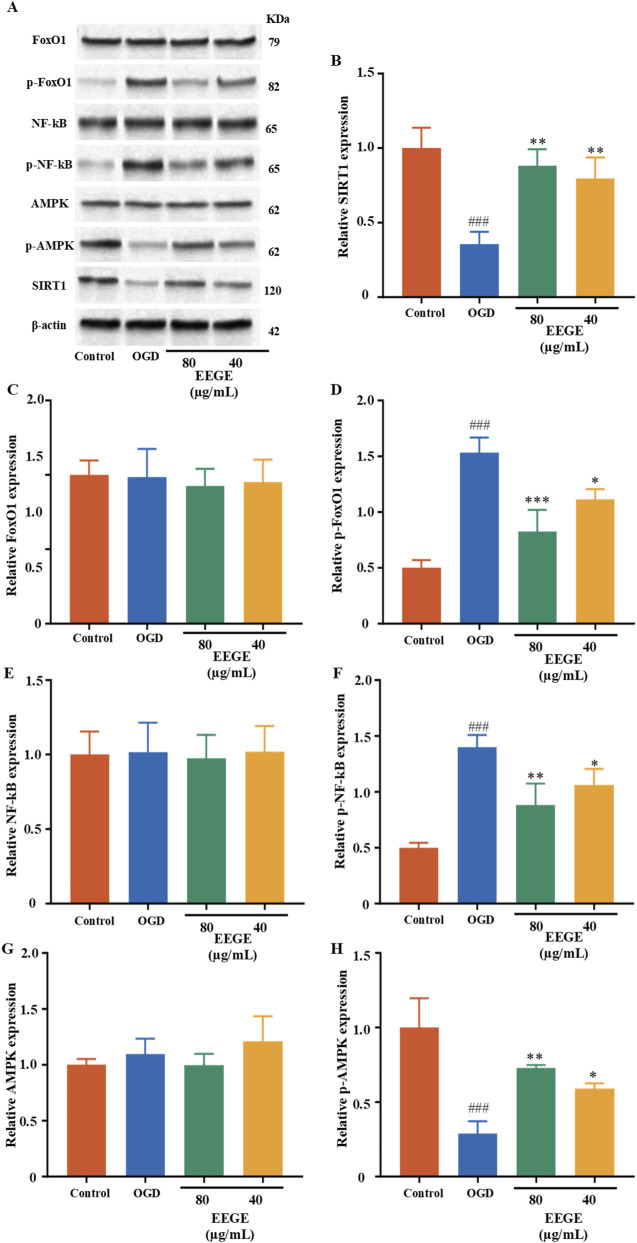
Western blot analysis in HT22 cells. **(A)** Protein band blot images and **(B–H)** respective statistical analysis of SIRT1, FoxO1, p-FoxO1, NF-κB, p-NF-κB, AMPK, and p-AMPK proteins. Significant differences were specific to the phosphorylated forms of the proteins (p-FoxO1, p-NF-κB, p-AMPK). Presented as mean ± SD (n = 3, biological replicate). vs. sham group, ^###^P < 0.001; vs. model group, *P < 0.05, **P < 0.01, ***P < 0.001.

We detected the expressions of key proteins in the IGF1-TREM2 signaling axis. The research results showed that, compared with the sham group, the protein levels of IGF-1 and Trem2 decreased (P < 0.001). Compared with the model group, EEGE re-versed the expressions of IGF-1 and Trem2 (P < 0.01, P < 0.05, Figures 13A–C). The results of protein expressions in the AMPK-SIRT1-FoxO1-NF-κB signaling pathway showed that, compared with the sham group, the protein levels of p-FoxO1and p-NF-κB increased, while the protein levels of p-AMPK and SIRT1 decreased (P < 0.001). After administration, these protein levels were significantly restored (P < 0.001, P < 0.01, P < 0.05, Figures 14A–H). There were no significant differences in non-phosphorylated proteins, and they were not statistically significant.

## 4 Discussion

VD is considered to be a cognitive disorder caused by cerebral vascular injury. Such damage may be caused by brain damage, stroke and other events, which in turn leads to cognitive function defects including reasoning, planning, judgment and memory (Hosoki et al., 2021; Morgan and Mc Auley, 2024; Song, 2024). Due to its complex etiology, effective pharmacological agents for the prevention and treatment of VD are still scarce (Reuben et al., 2024). This study systematically explores the potential mechanism of the beneficial effect of EEGE on VD by integrating traditional Chinese medicine metabolomics, network analysis, multi-omics technology and molecular biology experiments.

Initially, we established a 2-VO-induced VD rat model to simulate chronic cerebral hypoperfusion injury (Lamichhane et al., 2025). The results showed that 2-VO rats showed obvious neurological function defects, a significant decrease in cerebral blood flow, and significant pathological damage in the hippocampus region. In the behavioral assessments, 2-VO rats showed a significantly prolonged escape incubation period and increased swimming distance in the Morris water maze test. At the same time, the number of platform crossings was significantly reduced, suggesting that the spatial learning and memory functions were seriously impaired. These results confirm the successful replication of the chronic cerebral hypoperfusion model (Sun T. et al., 2023; Ningning et al., 2024). EEGE intervention significantly reversed the nerve function defect score, restored cerebral blood flow, improved the pathological damage of hippocampus tissue, and improved the spatial learning and memory ability of 2-VO rats, providing preliminary evidence of its anti-VD pharmacological effects.

We employed an integrated approach combining traditional Chinese medicine metabolomics, network analysis, and multi-omics detection data for predictive analysis. We systematically screened the potential interaction network between EEGE metabolites and VD-related targets. Through this analysis, we identified Parishin A and p-hydroxybenzaldehyde as the prioritized metabolites among the top 10 for assessment. This is because Parishin A is a quality control metabolite specified in the 2025 edition of the Chinese Pharmacopoeia, ensuring its relevance and importance to the medicinal material used. p-hydroxybenzaldehyde is a well-recognized metabolite of *G. elata*. Most importantly, our research group previously found that these two metabolites can effectively downregulate the expression of apoptosis-related proteins, reduce neuronal apoptosis, and exert significant neuroprotective effects.

By integrating metabolite-target analysis with plasma metabolomics and brain tissue transcriptomics, two key targets, TNF and IGF1, were identified. TNF serves as a critical initiating cytokine in the inflammatory response. During cerebral ischemia-hypoxia, even trace amounts of TNF can trigger and amplify inflammatory signaling pathways, stimulate immune cells, and thereby markedly upregulate the production and release of pro-inflammatory cytokines such as IL-6 and IL-1β. These cytokines accumulate in the lesioned areas, activating systemic inflammatory responses, exacerbating intracerebral inflammation, inducing hippocampal inflammation, leading to neuronal injury or death, impairing cognitive function, and ultimately contributing to the development of vascular dementia.

In the pathway analysis, we found that IGF1 showed significant changes in the AMPK pathway, which might be another core target for the treatment of VD by EEGE. At present, IGF-1, as a key protein in the AMPK pathway, has a wide range of non-specific neurotrophic effects, promoting neuronal regeneration and providing protection for the nervous system (Wu et al., 2017; Tian et al., 2022). As an important upstream regulatory factor of IGF1, TREM2 regulates the function of small glial cells, reduces the release of IL-1β and TNF-α, relieves neuroinflammation, and may delay the process of VD (Chen et al., 2020; Liu et al., 2022). AMPK is the main energy sensor and cellular energy homeostasis regulator. The activation of the AMPK signaling pathway promotes the expression of anti-inflammatory factors, thus producing anti-inflammatory effects (Bess et al., 2011; Mihaylova and Shaw, 2011; Wang et al., 2017; Qin and Song, 2022). In the omics assays, apart from IGF1, there were also significant changes in the expression of the FOXO gene in the AMPK pathway. A large amount of evidence shows that AMPK enhances the expression of SIRT1 and regulates FoxO activity, thus inhibiting the pro-inflammatory transcription factor NF-κB and reducing neuroinflammatory damage (Duan et al., 2019; Wang X. D. et al., 2021; Cai et al., 2022).

Based on our integrated network analysis and multi-omics analysis, we hypothesized that EEGE alleviates neuroinflammation and exerts anti-VD effects potentially through activation of the AMPK/SIRT1/FoxO1/NF-κB signaling pathway and the TREM2/IGF1 signaling axis. *In vivo* experimental results demonstrated that, compared to the sham group, the model group exhibited decreased protein expression of p-AMPK/AMPK, SIRT1, TREM2, and IGF1, whereas the ratios of p-FoxO1/FoxO1 and p-NF-κB/NF-κB increased. EEGE intervention significantly reversed these protein expression trends, with the high-dose group showing the most pronounced effects. Furthermore, EEGE significantly reduced abnormally elevated serum levels of inflammatory cytokines, including those of TNF, and IL-1β, in model rats. *In vitro* experiments further corroborated that EEGE improves the survival of HT22 cells under OGD injury by modulating the AMPK/SIRT1/FoxO1/NF-κB, and TREM2-IGF1 signaling axes.

## 5 Conclusion

EEGE mainly activates the TREM2-IGF1 neuroprotective axis through its key active metabolite Parishin A and p-hydroxybenzaldehyde, and regulates the AMPK/SIRT1/FoxO1/NF-κB signaling pathway. This dual action inhibits neuroinflammation while promoting neural repair and homeostasis, ultimately ameliorating VD-associated cognitive impairment. This study systematically revealed the multi-metabolite, multi-target, and multi-pathway characteristics of EEGE against VD, providing a theoretical foundation for its potential application in VD treatment.

## Data Availability

The datasets presented in this study can be found in online repositories. The names of the repository/repositories and accession number(s) can be found below: https://www.ebi.ac.uk/metabolights/MTBLS13695, https://www.ncbi.nlm.nih.gov/bioproject/PRJNA1403482.
